# A secondary analysis of cortical atrophy and plasma amyloid β patterns in older patients with cognitive frailty undergoing elective surgery

**DOI:** 10.1186/s12877-025-05740-z

**Published:** 2025-07-02

**Authors:** Rudolf Mörgeli, Friedrich Borchers, Insa Feinkohl, Sophie K. Piper, Tobias Pischon, Arjen J.C. Slooter, Claudia Spies, Janine Wiebach, Georg Winterer, Norman Zacharias, Florian Lammers-Lietz

**Affiliations:** 1 Department of Anesthesiology and Intensive Care Medicine| CCM| CVK, Charité – Universitätsmedizin Berlin, corporate member of Freie Universität Berlin, Humboldt- Universität zu Berlin, Augustenburger Platz 1, 13353 Berlin, Germany; 2 Berlin Institute of Health at Charité – Universitätsmedizin Berlin, Charitéplatz 1, 10117 Berlin, Germany; 3 Molecular Epidemiology Research Group, Max-Delbrück-Center for Molecular Medicine in the Helmholtz Association (MDC), Berlin, Germany; 4 Faculty of Health at Dept. of Medicine, Witten/Herdecke University, Witten, Germany; 5 Institute of Biometry and Clinical Epidemiology, Charité – Universitätsmedizin Berlin, corporate member of Freie Universität Berlin, Humboldt- Universität zu Berlin, Charitéplatz 1, 10117 Berlin, Germany; 6 Institute of Medical Informatics, Charité – Universitätsmedizin Berlin, corporate member of Freie Universität Berlin, Humboldt- Universität zu Berlin, Charitéplatz 1, 10117 Berlin, Germany; 7 Biobank Technology Platform, Max-Delbrück-Center for Molecular Medicine in the Helmholtz Association (MDC), Berlin, Germany; 8 Departments of Psychiatry and Intensive Care Medicine and Brain Center UMC Utrecht, University Medical Center Utrecht (UMC), Utrecht University, Utrecht, the Netherlands; 9 Department of Neurology, UZ Brussel and Vrije Universiteit Brussel, Brussels, Belgium; 10 Pharmaimage Biomarker Solutions Inc, Cambridge, MA USA; 11 PI Health Solutions GmbH, Berlin, Germany; 12 Department of Otorhinolaryngology, Head and Neck Surgery, Charité – Universitätsmedizin Berlin, corporate member of Freie Universität Berlin, Humboldt- Universität zu Berlin, Hindenburgdamm 30, 12203 Berlin, Germany

**Keywords:** Frailty, Cognitive frailty, Aging, MRI, Cortical atrophy, Amyloidβ

## Abstract

**Background:**

The etiology of cognitive impairment in frailty may be related to age or to an independent neurodegenerative process, such as Alzheimer’s disease (AD). In this secondary analysis, we examine cognitive frailty in patients aged 65 and older undergoing elective surgery, and explore associations with aging- and AD-related cortical atrophy patterns and amyloid β (Aβ) concentrations.

**Methods:**

Cognitive frailty (CF) was defined as the co-occurrence of (pre-)frailty and cognitive impairment (CI). Cognitive performance was assessed using the MMSE and the Cambridge Neuropsychological Teste Automated Battery (CANTAB), while frailty was assessed with a modified version of Fried’s frailty phenotype. Aging- and AD-related cortical atrophy patterns were derived from T1-weighted MRI using Freesurfer software. MRI patterns and plasma concentrations of Aβ species 40 and 42 (including Aβ 42/40 ratio) were compared to physically robust, cognitively unimpaired patients using multiple regression analyses and presented as regression coefficient b with 95% confidence intervals.

**Results:**

MRI data of *N* = 489 patients (*N* = 251 with frailty, *N* = 15 with CI, *N* = 43 with CF) and plasma Aβ concentrations of *N* = 786 patients (*N* = 400 with frailty, *N* = 20 with CI, *N* = 101 with CF) were analyzed. Cognitive frailty was associated with both aging-related and AD-related MRI signatures (b_age_=-0.070 [-0.113; -0.028], b_AD_=-0.069 [-0.118; -0.020]). Amyloid β42 was significantly lower in frail patients (b=-0.14 [-0.29; -0.01]), while β42/β40-ratio was lower in patients with frailty (b=-0.11 [-0.21; -0.01]) and cognitive frailty (b=-0.015 [-0.28; -0.03]).

**Conclusion:**

Our results suggest that atrophy in aging- and AD-related cortical regions is associated with cognitive frailty. Plasma amyloid β42/β40-ratios were significantly lower in patients with frailty and cognitive frailty, suggesting that (pre-)frailty in general, rather than cognitive frailty specifically, is associated with AD-like changes. Hence, AD-related pathology seems to be associated with cognitive frailty, but the available data is not sufficient to indicate shared pathomechanisms between AD and cognitive frailty.

**Trial Registration:**

ClinicalTrials.gov. Identifier NCT02265263, Date October 15th, 2014.

**Supplementary Information:**

The online version contains supplementary material available at 10.1186/s12877-025-05740-z.

## Introduction

Frailty syndrome is a condition related to a reduction of functional reserves and compensatory capacity, rendering individuals vulnerable to homeostatic disturbances [1]. Although frailty can develop at any age, older patients are more likely to be affected due to an accumulation of deficits and comorbidities [2]. Despite a strong focus on physical capacity and function, frailty is a multidimensional syndrome that also encompasses cognitive, social, and psychological domains. Much like physical frailty, cognitive impairment can severely impact quality of life and contribute to a loss of autonomy, with significant personal and social consequences.

The concurrent presentation of physical frailty and cognitive impairment has been termed cognitive frailty, and there has been considerable debate on how the pathogeneses of these conditions might be related [3]. The International Academy on Nutrition and Aging (I.A.N.A.) and the International Association of Gerontology and Geriatrics (I.A.G.G.). consensus definition of cognitive frailty excludes its co-occurrence with dementia, and in fact, existing literature supports the hypothesis of a selective impairment of frontal executive functions in cognitive frailty [4]. Nevertheless, physical frailty has been found to be associated with the development of mild cognitive impairment (MCI) and Alzheimer’s dementia (AD) [5, 6, 7], and it remains unknown whether cognitive frailty is an independent pathological entity or a coincidental presentation of preclinical dementia and physical frailty.

With the introduction of anti-amyloid drugs for AD [8], it becomes increasingly important to identify not only patients that are vulnerable to amyloid-related neurocognitive decline, but also those that would benefit most from these specific therapies. Hence, it is essential to understand the overlap between AD, frailty and cognitive frailty, which are highly interrelated, but per definition exclusive. Studies on AD-related biomarkers in cognitive frailty are needed to re-define the borders between these conditions.

Frailty has been found to be associated with brain pathology in both post-mortem [9, 10] and in-vivo magnetic resonance imaging (MRI) studies [11, 12, 13, 14]. Although decline in cognition and progression of frailty have been found to be associated, neuropathology contributes far more to the former than to the latter [9]. Recent studies have attributed structural brain alterations in frailty to vascular comorbidity rather than to primary neurodegenerative disease [11, 12]. In contrast, an association between aging-related brain atrophy and cognitive impairment has been repeatedly described in the literature [15, 16, 17].

Dickerson and colleagues described a specific pattern of cortical thinning in patients with Alzheimer’s dementia, which was found to be associated with cognitive symptoms in patients at the earliest stage of disease, as well as β-amyloid deposition in asymptomatic patients [18]. The same research group later found that this cortical AD signature predicted progression to AD in patients with early signs, suggesting that this atrophy pattern is present in patients with AD prior to clinical manifestation [19]. In a subsequent work, they defined another distinct aging-related cortical signature that was associated with cognitive function in healthy older adults [20]. The terms frailty and aging are not interchangeable, but frailty prevalence increases with age [2]. Although it remains unclear whether aging-related brain atrophy contributes to the development of frailty, it has been shown that sensorimotor areas (e.g. precentral gyrus, insula, and occipital cortex) are affected [20], predisposing patients to motor impairment and physical frailty, as our previous work suggests [21]. Conversely, the aging signature affects attention-related dorsomedial and inferior frontal cortical areas, which may lead to cognitive deficits in frontal executive functions, which were previously described in frail individuals [4, 22]. Both signatures have been linked to several other clinical conditions associated with cognitive and motor symptoms, such as memory function across the age range, postoperative delirium, and cognitive dysfunction, as well as Parkinson’s disease and Lewy body dementia [23, 24, 25, 26].

Aside from pathognomonic brain structural changes, molecular markers of Alzheimer’s disease, such as amyloid β, have been investigated in frail patients. To our knowledge, no study has addressed peripheral amyloid β in cognitive frailty, nor its association with aging- and AD-related cortical atrophy patterns.

The prevalence of (pre-)frailty differs among populations, with the lowest prevalence observed in population-based studies, and a higher prevalence in hospitalized patients, which may be linked to the cause of hospital admission [27, 28]. Hence, etiologies for cognitive frailty in hospitalized patients are expected to differ from the general population. Understanding frailty in surgical patients is of particular interest, as frailty is associated with overall higher rates of postoperative complications [29]. Currently, no studies have examined amyloid β and cortical atrophy in surgical patients.

This secondary analysis investigates cortical thinning signatures and plasma β-amyloids Aβ40 and Aβ42 in a sample of surgical patients without apparent dementia, and examines whether AD-related neuropathology correlates with cognitive frailty [18, 30]. We hypothesize that cortical thinning in selected AD- and aging-related brain regions, as well as plasma β-amyloids levels, differ among robust patients, patients with either cognitive impairment or frailty alone, and patients with cognitive frailty. Our study aims to improve our biomedical understanding of cognitive frailty, and how it could contribute to the increased vulnerability of surgical patients. Additionally, an exploratory analysis examines the association of cortical signatures with β-amyloid concentration as blood-based biomarkers for neurodegenerative disease.

## Methods

### Study design and procedures

This is a secondary analysis of data collected in the BioCog project (Biomarker Development for Postoperative Cognitive Impairment in the Elderly study, www.biocog.eu), a prospective multicenter cohort study aimed at developing a biomarker-based algorithm for risk prediction of postoperative cognitive disorders [31]. Patients ≥ 65 years of age, of European-Caucasian descent presenting for elective major surgery (≥ 60 min) were recruited in Berlin, Germany, and Utrecht, Netherlands between October 2014 and December 2017. Exclusion criteria comprised lack of consent, Mini-Mental State Examination (MMSE) score ≤ 23 points, neuropsychiatric morbidity, centrally acting medication, sensory impairment interfering with neurocognitive testing or MRI, homelessness, or unavailability of the patient for follow-up assessments, simultaneous participation in another prospective interventional clinical trial, and accommodation in an institution due to an official or judicial order.

Inclusion and exclusion criteria in the BioCog were chosen with respect to the primary aim of predicting postoperative delirium and cognitive decline from multimodal data. Hence, patients ≥ 65 years of age with major surgery were included to ascertain a relevant risk for postoperative delirium. European-Caucasian descent was required to achieve a genetically homogeneous sample that allowed analysis of genetic risk factors. Patients with major cognitive impairment or sensory impairment that might jeopardize neuropsychologic testing were excluded. Patients with neuropsychiatric disease or centrally acting medication were excluded to avoid bias in delirium assessments and functional neuroimaging.

Written informed consent for participation was obtained from all patients prior to inclusion. All procedures were approved by the local ethics committees in Berlin, Germany (EA2/092/14) and Utrecht, Netherlands (14–469) and conducted in accordance with the declaration of Helsinki. The study was registered under the identifier NCT02265263 at clinicaltrials.gov on 15/10/2014.

### Frailty assessment

Frailty was defined according to a modified version of Fried’s Physical Phenotype, as previously described [11, 12], assessing the criteria slowness, weakness, exhaustion, weight loss and low activity [1]. The assessment took place within two weeks of surgery, and patients were deemed pre-frail if one or two criteria were fulfilled, and frail if three or more criteria were present. This analysis did not differentiate between pre-frailty and frailty.

All items were measured prospectively, but modifications to Fried’s original score were necessary to suit the German cohort and the available data. Although this modified version has not been validated, previous publications show that this score is sensitive to brain atrophy and cerebral vessel disease, as well as functional connectivity of the supplementary motor cortex [11, 12, 21].

### Cognitive assessment

Patients were screened for major cognitive impairment at inclusion using the MMSE. Additional cognitive testing took place prior to surgery using a comprehensive test battery consisting of a screen-based neuropsychological test (CANTAB, Cambridge Cognition Ltd., Cambridge, UK) and paper-and-pencil tests (Trail-Making-Test Parts A and B). A detailed description of the neuropsychological testing is provided in the supplement.

Finally, a dichotomous indicator variable for preexisting cognitive impairment was calculated using a previously described procedure, which was referred to as PreCI (preoperative cognitive impairment) in earlier publications [32, 33]. In short, z-scores of the baseline measurement were calculated for each test parameter in a non-surgical reference dataset, and the same z-transformation was then applied to the sample dataset. Two z-scores below − 1.96 in single cognitive test parameters or a compound z-score below − 1.96 averaged over all z-scores was used to define cognitive impairment [33]. Additional computational details are available in the code for the POCDr package for R (https://github.com/Wiebachj/POCDr/).

For the purposes of this analysis, cognitive impairment was defined as results below-reference performance in the neuropsychological battery and/or an MMSE score ≤ 25 during the study eligibility screening (further description below).

### Definition of cognitive frailty

In 2013, an international consensus group organized by the International Academy on Nutrition and Aging (I.A.N.A.) and the International Association of Gerontology and Geriatrics (I.A.G.G.) defined cognitive frailty as the combination of physical frailty and cognitive impairment in the absence of dementia. While a precise adaptation of the I.A.N.A./I.A.G.G. definition was not possible in the current investigation due to the design of the BioCog study and the available dataset, we attempted to harmonize the definition of cognitive frailty used here with I.A.N.A./I.A.G.G. criteria. The consensus group recommended operationalizing cognitive impairment as a Clinical Dementia Rating (CDR) of 0.5 (very mild dementia) [3]. This definition was adapted to allow analysis of the available data, as the CDR had not been assessed in the BioCog study [31]. Beyond I.A.N.A./I.A.G.G. criteria, various definitions of cognitive impairment have been used in cognitive frailty research [34], and among those using the MMSE, a cut-off of 25 points or less has been established [34, 35, 36]. A previous study on the German version of the MMSE also indicated that a score of 21–25 points corresponds to a CDR of 0.5 [37]. Hence, cognitive impairment was defined by either below-reference performance in the CANTAB testing, as described above, and/or an MMSE score ≤ 25 points during the study eligibility screening. While the I.A.N.A./I.A.G.G. criteria require the full presentation of physical frailty as a prerequisite for cognitive frailty (i.e., fulfilling ≥ 3/5 of Fried’s criteria), we accepted the presence of at least one of Fried’s criteria (corresponding to pre-frailty) for our definition. This adaptation was necessary due to the low number of patients fulfilling at least three criteria, as well as the fact that pre-frailty is already associated with a relevant increase in poor outcomes in surgical cohorts [29]. Finally, cognitive frailty was defined as the combination of (pre-)frailty and cognitive impairment. In this article, the term “cognitive frailty” refers to patients with both physical frailty and cognitive impairment, whereas the terms “cognitive impairment” and “frailty” are reserved for patients with only one of these two conditions, respectively.

### Determination of cortical atrophy

#### Magnetic resonance imaging

T1-weighted MR imaging was conducted within 2 weeks of all other assessments. Cortical volumetry was performed using the fully automated parcellation pipeline available from the FreeSurfer software (http://surfer.nmr.mgh.harvard.edu)^17^. Volumetric data for all regions defined in the Desikan-Killiany (DK) atlas [38] are available from the BioCog consortium.

#### Cortical atrophy pattern

Three previously described volumetric parameters were derived: an AD signature, an aging signature, and the personalized AD index (pADi), which is the ratio of the former two. Unlike previous studies, which used maps generated from comparisons of AD patients with healthy controls [18, 39], this analysis used labels from the Desikan-Killiany atlas – an approach that has been successfully utilized previously [26]. To assess confounding by global cortical atrophy, mean cortical thickness was also analyzed.

### Amyloid β

Blood samples were collected immediately prior to surgery, on the first postoperative day, and three months after surgery, whereas only the preoperative samples were used in this investigation. Blood was collected by trained clinical staff according to a standard operating procedure. Blood samples were frozen at -80˚C and shipped to a central laboratory in Berlin for sample processing and biobank storage, as well as analysis of further parameters at the Molecular Epidemiology Group, Max-Delbrück Center (MDC), Berlin. Aβ42 and Aβ40 were measured using two specific ELISA sandwich kits, ABtest40 and ABtest42, by Araclon Biotech in Zaragoza, Spain [40].

### Statistical analysis

We describe the sample stratified by groups of patients (control patients, (pre-)frail and cognitively impaired patients, and patients with cognitive impairment) using median and quartiles for continuous and ordinal data, and absolute and relative frequencies for nominal data. Differences among groups were tested for significance using either Χ²-test or Kruskal-Wallis-test (with Dunn’s test for pairwise comparisons for cortical signatures and amyloid levels).

Multiple linear regression was used to assess effects of cognitive frailty on cortical atrophy in the primary analyses. Aging and AD signatures, as well as the personal AD index were found to be normally distributed, and hence, analyzed as dependent variables in three multiple ordinary least squares linear regression models (primary models). In these, cognitive frailty, frailty and cognitive impairment were coded as three dummy variables and compared to cognitively unimpaired, physically robust preoperative surgical patients (reference category). Sex, age, and two dummy variables for scanner type were included in all models as confounding variables.

Aβ40 and Aβ42 levels, as well as the Aβ42/Aβ40-ratio were found to be heavily right-skewed. Hence, three generalized linear models were employed for Aβ40, Aβ42, and their ratio, assuming a gamma distribution with a logarithmic link function, as discussed in previous publications on analysis of right-skewed data [41, 42], including amyloid deposition [43]. Cognitive frailty, frailty and cognitive impairment were again coded as three dummy variables and compared to cognitively unimpaired, physically robust patients (reference category). Models were adjusted for age, sex and a nuisance variable related to amyloid measurements describing the ELISA batch.

Regression analysis results are presented as regression coefficients with 95% confidence intervals (95% CI) based on 100 000 bootstrap samples. In a control model, mean cortical thickness was analyzed using the same model specifications to evaluate if AD and aging signatures were specifically associated with cognitive frailty or just reflected global, unspecific cortical thinning. Primary models and the control model were compared with respect to adjusted R² and Akaike’s information criterion (AIC). To compare generalized linear models, we calculated D² (D^2^=(Null deviance – Residual Deviance)/Null Deviance) instead of R², as described previously [16].

Due to the low number of non-frail patients with cognitive impairment and the associated statistical uncertainty, there was considerable difficulty in discriminating between atrophy and amyloid changes associated specifically with cognitive frailty and a more general association with cognitive impairment in both physically frail and non-frail patients. Hence, our primary models were compared to alternative models, including frailty and cognitive impairment as independent variables (without a term for cognitive frailty). Primary and alternative models were compared using the J test and AIC.

Associations of cortical signatures and β-amyloid concentrations were analyzed using Spearman’s rank correlation coefficient (ρ).

*P* < 0.05 was set as the general level of significance. All *p*-values constitute exploratory analyses and do not allow for confirmatory generalization of results. All analyses were performed with R v4 using the stats, car, and boot packages.

Since this is a secondary analysis, no a-priori power calculations have been performed.

## Results

### Sample description

**Fig. 1:**
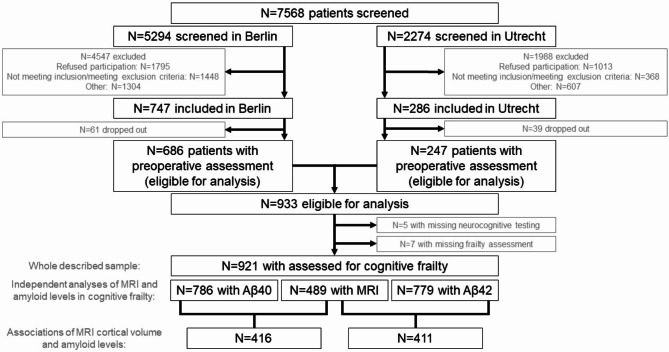
The patient flow chart

Figure 1 depicts the study screening and recruitment at each stage, and number of patients included in the presented analyses. 933 patients were included in the BioCog study, and 928 patients underwent neurocognitive testing (CANTAB). Of these, 921 had a valid frailty assessment, and 489 of them had an additional T1w structural MRI and could be included in this investigation. Plasma amyloid β40 and amyloid β42 levels were collected from 786 to 779 patients, respectively.

Of 489 patients with MRI data, 259 (53%) patients met at least one criterion of Fried’s modified frailty phenotype, 12 (3%) patients fulfilled criteria for isolated cognitive impairment according to the compound definition (based on CANTAB assessments and/or an MMSE score ≤ 25 points), and 43 (9%) fulfilled criteria for cognitive frailty. 175 (36%) patients were physically robust and cognitively healthy. Among patients with frailty (without cognitive impairment), Fried’s score ranged between 1 and 4 (median 1, interquartile range 1–2), and among patients with cognitive frailty, the score ranged between 1 and 4 (median 2, interquartile range 1–3). In the group of patients with cognitive impairment (without frailty), 12 patients were allocated due to CANTAB assessment criteria, and three due to MMSE criteria. None of these patients fulfilled both criteria. Among patients with cognitive frailty, 29 patients had cognitive impairment according to CANTAB assessment only, and eight patients were identified in the MMSE. Six cognitively frail patients showed impairment in both CANTAB and MMSE. Table 1 provides a detailed description of the patients with available MRI for cortical signature analysis.

**Table 1:** Description of the study sample for analysis of cortical signatures. Continuous variables are described as median, interquartile range (min.-max. range), and categorical variables as absolute number (relative frequency in %)

	Control* **(N = 180)**	(Pre-)frailty (N = 251)	Cognitive impairment (N = 15)	Cognitive frailty (N = 43)	Χ² (p)**
Aging signature	2.57, 2.49–2.68 (2.10–2.89)	2.56, 2.47–2.64 (2.14–3.02)	2.52, 2.49–2.57 (2.33–2.78)	2.51, 2.42–2.57^+^ (2.20–2.74)	***10.7*** ***(0.014)***
AD signature	2.69, 2.61–2.80 (2.18–3.02)	2.68, 2.58–2.77 2.21–3.14	2.66, 2.63–2.75 (2.47–2.88)	2.64, 2.53–2.71 (2.30–2.95)	6.82 (0.078)
Personalized AD index	9.56, 9.44–9.68 (9.01–10.10)	9.56, 9.38–9.70 (8.97-10.00)	9.48, 9.40–9.66 (9.13–9.95)	9.52, 9.33–9.60 (9.12–9.87)	2.43 (0.488)
Age (y)	70, 67–73 (65–87)	73, 69–76^+^ (65–87)	71, 68–75 (66–77)	74, 69–77^+^ (65–85)	***23.9*** ***(0.001)***
Women	48 (27%)	111 (44%)	2 (13%)	29 (67%)	***33.4*** ***(< 0.001)***
MMSE (p)	29, 28–30 (26–30)	29, 28–30 (26–30)	28, 26–29^+,++^ (24–30)	28, 25–28^+^ (24–30)	***48.4*** ***(0.001)***
CCI (p)	1, 0–2 (0–8)	1, 0–2^+^ (0–9)	1, 0–2 (0–4)	1, 0–2^+^ (0–6)	***8.7*** ***(0.033)***
Arterial hypertension	105 (58%)	157 (63%)	10 (66%)	28 (65%)	1.7 (0.630)
CAD	33 (18%)	48 (19%)	1 (7%)	8 (19%)	1.3 (0.726)
Diabetes mellitus	29 (16%)	64 (25%)	4 (27%)	11 (26%)	6.2 (0.104)
Stroke or TIA	8 (4%)	27 (11%)	2 (13%)	9 (21%)	***15.6*** ***(0.017***)
Malignant disease	39 (22%)	71 (28%)	5 (33%)	10 (23%)	3.8 (0.288)
GDS (p)	1, 0–1 (0–4)	2, 1–3^+,^ (0-12.9)	1.1, 1–3^+^ (0–8)	2.1, 1-5.8^+,++^ (0–11)	***74.1*** ***(< 0.001)***
EQ-5D	1.0, 0.8-1.0 (0.6-1.0)	0.8, 0.7-1.0^+^ (0.2-1.0)	1.0, 0.9-1.0^++^ (0.8-1.0)	0.8, 0.6–0.9^+,++,+++^ (0.4-1.0)	***35.9*** ***(< 0.001)***
IADL (p)^##^	8, 8–8 (7–8)	8, 8–8^+^ (1–8)	8, 8–8 (8–8)	8, 7–8^+,++,+++^ (5–8)	***26.*** ***(< 0.001)***
Barthel score (p)^##^	100, 100–100 (85–100)	100, 100–100^+^ (55–100)	100, 100–100 (95–100)	95, 92.5–100^+,++,+++^ (70–100)	***32.2*** ***(0.001)***
MNA: at risk	16 (9%)	58 (23%)	2 (13%)	10 (23%)	***38.0*** ***(< 0.001)***
MNA: malnourished	0 (0%)	21 (8%)	0 (0%)	2 (5%)	
BMI (kg/m²)	26.4, 24.0-28.5 (17.9–35.9)	26.7, 24.3–29.5 (17.6–43.1)	26.8, 23.2–27.3 (16.9–35.2)	27.2, 23.2–32.0 (14.7–44.3)	4.1 (0.256)
Secondary education^#^	53 (29%)	84 (33%)	4 (27%)	21 (49%)	***22.7*** ***(< 0.001)***
Tertiary education^#^	84 (46%)	95 (38%)	5 (33%)	5 (12%)	
Albumin (g/L)^§^	41.3, 38.7–43.8 (15.5–51.6)	40.6, 38.4–42.7 (22.2–48.7)	41.4, 38.2–43.9 (21.8–45.5)	41.9, 37.1–44.1 (33.1–48.6)	4.9 (0.176)
Creatinine (µmol/L)^§^	77.9, 69.8–91.0 (32.7–160.0)	74.3, 62.8–86.8 (38.0-529.5)	77.3,70.3-109.8 (54.8–112.0)	73.7, 69.8–91.0 (47.7-123.8)	4.9 (0.177)
NT-proBNP (pmol/L)^§,###^	6.1, 2.9–16.0 (2.9-617.2)	6.7,2.9–23.8 (2.9-367.3)	2.9, 2.9-4.0 (2.9–15.4)	9.4, 2.9–33.8 (2.9-397.8)	4.70 (0.195)
Hemoglobin (g/dL)^§^	13.7, 12.7–14.5 (5.4–17.0)	12.9, 11.7–14.1^+^ (7.0-17.9)	14.2, 12.3–14.5 (10.1–16.3)	12.7, 11.0-14.2 ^+^ (8.1–16.2)	***20.19*** ***(< 0.001)***

* The term “control” refers to the group of physically robust patients without cognitive impairment.

** Results from a Kruskal-Wallis-test with three degrees of freedom for continuous and ordinal data and a Χ²-test with three degrees of freedom for nominal data

^+^ Significantly different from control patient group (*p* < 0.05, unadjusted, Dunn’s test)

^++^ Significantly different from (pre-)frail patient group (*p* < 0.05, unadjusted, Dunn’s test)

^+++^ Significantly different from cognitively impaired patient group (*p* < 0.05, unadjusted, Dunn’s test)

^#^ According to ISCED: level 1–2 – primary education, level 3–4 – secondary education, level 5 and above – tertiary education

^##^ See also supplementary figure S1

^###^ Values below the detection level (3pmol/L) were replaced with 2.9pmol/L.

^§^ Laboratory values are given to provide estimates for malnourishment and catabolic status (albumin), cachexia and muscular hypotrophy (creatinine), cardiac insufficiency (NT-proBNP) and anemia (hemoglobin) in our cohort

Abbreviations: CAD - Coronary artery disease; CCI - Charlson’s comorbidity index; EQ - 5D–Health–related quality of life (5 dimensions); GDS - Geriatric depression scale; IADL - Instrumental activities of daily living; MMSE - Mini–mental status examination; p - points; TIA - transient ischemic attack; y - years

Of the overall sample of 786 patients with plasma amyloid concentrations, 400 (51%) fulfilled at least one criterion for frailty, 20 (3%) were categorized as having an isolated cognitive impairment and 101 (13%) were found to be cognitively frail. 265 (34%) patients were robust and cognitively healthy. Among frail patients, Fried’s frailty score ranged between 1 and 4 (median 1, interquartile range 1–4), whereas among cognitively frail patients the score ranged between 1 and 5 (median 2, interquartile range 1–3). Of 20 patients with an isolated cognitive impairment, 2 were allocated to this group based on their MMSE, 17 based on CANTAB testing and 1 fulfilled both criteria. Of 101 cognitively frail patients, 18 had an MMSE ≤ 25, 67 showed cognitive impairment on CANTAB testing, and 16 fulfilled both criteria. Table 2 provides a detailed description of the sample with available amyloid β data. Table 3 describes the distribution of cortical signatures and amyloid β levels in the investigated cohort.

**Table 2:** Description of the study sample for analysis of plasma β amyloid concentrations (N = 786). Continuous variables are described as median, interquartile range (min.-max. range), and categorical variables as absolute number (relative frequency in %)

	Control* (N = 265)	(Pre-)frailty (N = 400)	Cognitive impairment (N = 20)	Cognitive frailty (N = 101)	Χ² p**
Aβ40 (pg/mL)	272, 233–323 (57–930)	278, 24–319 (73–704)	257, 234–286 (179.6-450.2)	293, 245–352 (108–727)	6.6 (0.084)
Aβ42 (pg/mL)	32.4, 25.1–44.7 (7.1–526.0)	35.1, 26.1–44.8 (7.6-314.1)	39.3, 34.2–46.5 (13.5–116.0)	35.81, 29.0-46.2 (3.4-331.6)	6.8 (0.079)
Aβ42/Aβ40-ratio	0.12, 0.10–0.16 (0.03–0.88)	0.13, 0.10–0.16 (0.03–0.63)	0.15, 0.12–0.20 (0.06–0.36)	0.12, 0.10–0.16 0.03–0.46	5.9 (0.115)
Age (y)	70, 67–74 (65–87)	72, 69–76^+^ (65–89)	72, 70–75 (66–84)	75, 70–78^+,++^ (65–91)	***46.7*** ***(< 0.001)***
Women	86 (32%)	181 (45%)	3 (15%)	58 (57%)	***27.7*** ***(< 0.001)***
MMSE (p)	29, 28–30 (26–30)	29, 28–30 (26–30)	27, 27–29^+,++^ (24–30)	27, 25–28^+,++^ (24–30)	***111.8*** ***(< 0.001)***
CCI (p)	1, 0–2 (0–8)	1, 0–2^+^ (0–9)	1, 1–2 (0–4)	2, 0–3^+,++^ (0–10)	***18.2*** ***(< 0.001)***
Arterial hypertension	154 (58%)	257 (64%)	10 (50%)	73 (73%)	7.1 (0.068)
CAD	52 (20%)	76 (19%)	1 (5%)	21 (21%)	2.7 (0.444)
Diabetes mellitus	33 (12%)	105 (26%)	5 (25%)	32 (32%)	***23.3*** ***(< 0.001)***
Stroke or TIA	0 (0%)	33 (8%)	2 (10%)	18 (18%)	***24.24*** ***(< 0.001)***
Malignant disease	79 (31%)	131 (33%)	9 (5%)	36 (36%)	3.7 (0.30)
GDS (p)	1, 0–1 (0-6.4)	2, 0–3^+^ (0-12.6)	1, 0.8-2 (0–8)	2, 1–3^+,++,+++^ (0-12.9)	***135.4*** ***(< 0.001)***
EQ-5D	1.0, 0.8-1.0 (0.2-1.0)	0.8, 0.7–0.9 (0.2-1.0)	1.0, 0.9-1.0 (0.8-1.0)	0.8, 0.6–0.9 0.2-1.0	***77.0*** ***(< 0.001)***
IADL (p)^##^	8, 8–8 (7–8)	8, 8–8^+^ (0–8)	8, 8–8 (8–8)	8, 7–8^+,++,+++^ (2–8)	***72.6*** ***(< 0.001)***
Barthel (p)^##^	100, 100–100 (85–100)	100, 95–100^+^ (55–100)	100, 100–100 (95–100)	100, 90–100^+,++,+++^ (60–100)	***54.4*** ***(< 0.001)***
MNA: at risk	24 (9%)	107 (27%)	3 (15%)	36 (36%)	***76.7*** ***(< 0.001)***
MNA: malnourished	0 (0%)	340 (85%)	0 (0%)	7 (7%)	
BMI (kg/m²)	26.3, 23.8–28.7 (16.3–40.2)	26.9, 24.6–30.1^+^ (15.6–44.3)	24.4, 22.9–26.8^++^ (16.9–29.1)	27.8, 23.9–32.0^+,+++^ (14.7–46.8)	***16.0*** ***72.6*** ***(0.001)***
Secondary education^#^	89 (34%)	151 (38%)	9 (45%)	48 (48%)	***33.5*** ***(< 0.001)***
Tertiary education^#^	120 (45%)	146 (37%)	4 (20%)	13 (13%)	
Albumin (g/L)^§^	41.3, 38.4–43.7 (15.5–51.6)	40.6, 37.9–42.8^+^ (22.2–51.7)	41.3, 36.9–43.1 (21.8–45.5)	38.4, 32.3–43.4^+,++^ (22.1–48.7)	***16.4*** ***(< 0.001)***
Creatinine (µmol/L)^§^	78.3, 67.2–91.0 (32.7-160.9)	74.3, 63.2–88.2 (35.4-529.5)	77.3, 70.3–82.4 (49.5–112.0)	80.4, 63.7–93.7 (38.0-145.0)	3.2 (0.360)
NT-proBNP (pmol/L)^§,###^	6.1^#^, 2.9–14.9 2.9^#^-617.2	6.3, 2.9^#^-22.8 2.9^#^-367.3	3.5, 2.9–12.5 2.9–24.6	8.7, 2.9–35.1 2.9-397.8	3.5 (0.326)
Hemoglobin (g/dL)^§^	13.7, 12.6–14.5 5.4–17.9	13, 11.7–14.2^+^ (7.0-17.9)	14.1, 12.2–14.7 (10.1–16.3)	12.6, 11.5–13.9^+,+++^ (7.9–16.5)	***33.7*** ***(< 0.001)***

* The term “control” refers to the group of physically robust patients without cognitive impairment.

** Results from a Kruskal-Wallis-test with three degrees of freedom for continuous and ordinal data and a Χ²-test with three degrees of freedom for nominal data

^+^ Significantly different from control patient group (*p* < 0.05, unadjusted, Dunn’s test)

^++^ Significantly different from (pre-)frail patient group (*p* < 0.05, unadjusted, Dunn’s test)

^+++^ Significantly different from cognitively impaired patient group (*p* < 0.05, unadjusted, Dunn’s test)

^#^ According to ISCED: level 1–2 – primary education, level 3–4 – secondary education, level 5 and above – tertiary education

^##^ See also supplementary figure S1

^###^ Values below the detection level (3pmol/L) were replaced with 2.9pmol/L.

^§^ Laboratory values are given to provide estimates for malnourishment and catabolic status (albumin), cachexia and muscular hypotrophy (creatinine), cardiac insufficiency (NT-proBNP) and anemia (hemoglobin) in the cohort

Abbreviations: CAD – Coronary artery disease; CCI – Charlson’s comorbidity index; EQ-5D – Health-related quality of life (5 dimensions); GDS – Geriatric depression scale; IADL – Instrumental activities of daily living; MMSE – Mini-mental status examination; p – points; TIA – transient ischemic attack; y – years;

**Table 3:** Description of cortical signatures and Aβ levels as median, mean interquartile (IQR) and minimum to maximum (min.-max.) range for the whole sample

	Median (mean)	IQR	Min.-max.
Aging signature (cm)	2.56 (2.56)	2.48–2.66	2.10–3.02
AD signature (cm)	2.68 (2.68)	2.59–2.78	2.18–3.14
Personalized AD index	9.56 (9.54)	9.4–9.68	8.97–10.10
Mean cortical thickness (cm)	2.34 (2.34)	2.28–2.41	1.97–2.65
Aβ40 (pg/mL)	277.4 (288.5)	237.9-325.1	56.6-930.7
Aβ42 (pg/mL)	34.47 (43.06)	26.16–45.07	3.43-525.99
Aβ42/Aβ40-ratio	0.124 (0.145)	0.097–0.161	0.032–0.878

According to results from Kruskal-Wallis-test and Χ²-test, patient groups differed by age, sex, MMSE, Charlson Comorbidity Index, incidence of diabetes mellitus, cerebrovascular incidents, albumin and hemoglobin levels, Geriatric Depression Scale score, functional level on IADL and Barthel index, nutritional status in MNA and BMI, educational level and quality of life (see Tables 1 and 2 for detailed results). Among the parameters of interest, the Kruskal-Wallis-test yielded a significant result for a significant group difference in the aging signature (Table 1).

### Cortical signatures

**Fig. 2:**
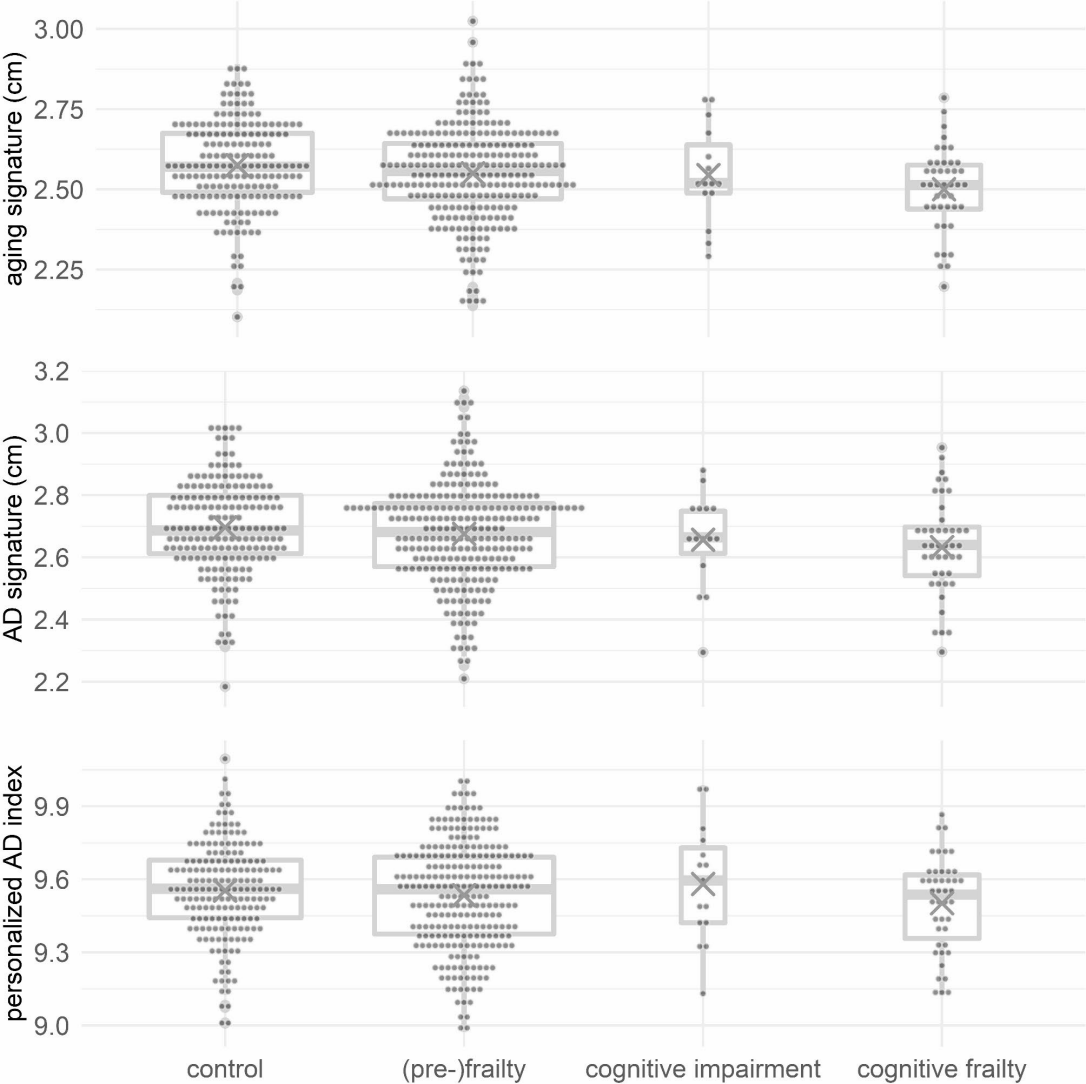
Boxplots with mean values (x) for cortical signatures

Aging signature: There was a significant negative association between cognitive frailty and the aging signature (b=-0.070 [-0.113; -0.028], *p* = 0.004), i.e., atrophy was more prominent in patients with cognitive frailty, but neither cognitive impairment (b=-0.024 [-0.102; 0.054], *p* = 0.5) nor (pre)frailty (b=-0.017 [-0.043; 0.010], *p* = 0.24) alone were associated with the aging signature (see Fig. 2, top, and supplementary table S1).

AD signature: There was a significant negative association of cognitive frailty and the AD signature (b=-0.069 [-0.118; -0.020], *p* = 0.010), i.e., atrophy was more prominent in cognitively frail patients, but neither cognitive impairment (b=-0.033 [-0.114; 0.041], *p* = 0.41) nor frailty (b=-0.015 [-0.044; 0.013], *p* = 0.32) were associated with the AD signature (see Fig. 2, middle, and supplementary table S2).

Personalized AD index: There were no differences among cognitive frailty (b=-0.014 [-0.079; 0.050], *p* = 0.7), cognitive impairment (b = 0.030 [-0.083; 0.140], *p* = 0.6) and frailty (b=-0.006 [-0.045; 0.032], *p* = 0.8) signature regarding the personalized AD index (see Fig. 2, bottom, and supplementary table S3).

Mean cortical thickness: A significant negative association was observed between mean cortical thickness with cognitive frailty (b=-0.055 [-0.083; -0.027], *p* = 0.001), indicating higher global brain atrophy in this group, but not with cognitive impairment (b=-0.025 [-0.075; 0.020], *p* = 0.307) or frailty (b=-0.013 [-0.030; 0.005], *p* = 0.172). Adjusted R² for this model was 0.169, which was considerably higher than for the other presented models (R²_age_=0.080, R²_AD_=0.095). Accordingly, AIC for the model of mean cortical thickness was − 2335, and hence also lower than the AICs for the other models (AIC_age_=-1926, AIC_AD_=-1845).

Since some patients had significant functional impairment, we suspected that patients with undiagnosed AD might have been included. Therefore, analyses for the personalized, aging- and AD-signatures were repeated in a subsample with Barthel score of ≥ 95 points and IADL score of 8. Results did not change substantially (see supplementary tables S8-S10).

### Plasma amyloid β concentrations

**Fig. 3:**
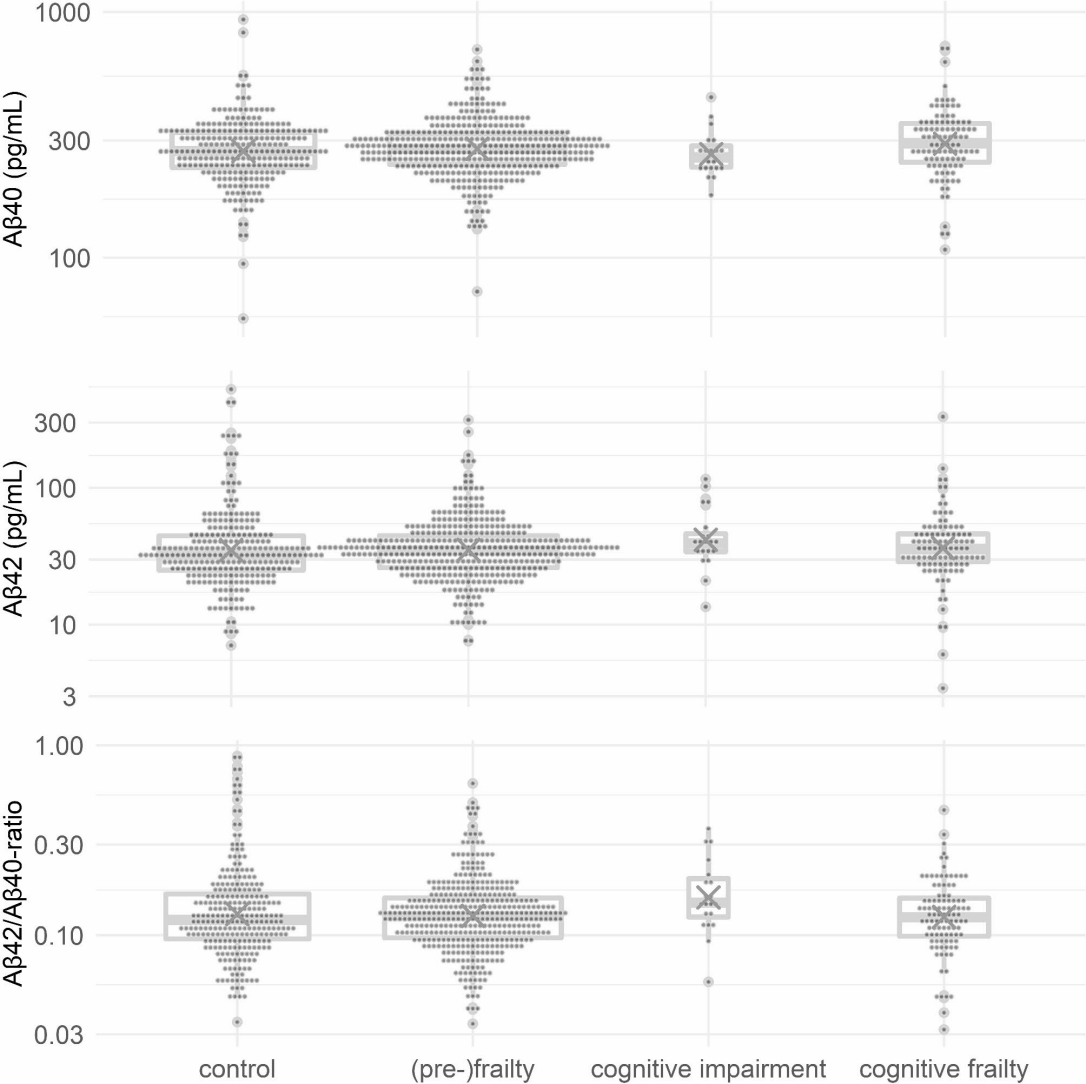
Boxplots with mean values (x) for blood-sampled for β-amyloid concentrations. Y-axis have been log-transformed for purposes of visualization

Aβ40 levels: At a non-significant trend level, cognitive frailty was associated with higher levels of serum Aβ40 (b = 0.07 [-0.01; 0.15], *p* = 0.067), but neither cognitive impairment (b=-0.04 [-0.16; 0.08], *p* = 0.524) nor frailty (b = 0.01 [-0.04; 0.06], *p* = 0.650) showed a relation to serum concentrations. Aβ40 levels increased over age (b = 0.006, *p* = 0.015, see Fig. 3, top, and supplementary table S5).

Aβ42 levels: Aβ42 levels were lower in patients with frailty (b=-0.14 [-0.29; -0.01], *p* = 0.044), and levels in those with cognitive frailty (b=-0.11 [-0.32; 0.11], *p* = 0.294) and cognitive impairment (b=-0.08 [-0.37; 0.19], *p* = 0.672) did not differ significantly (see Fig. 3, middle, and supplementary table S6).

Aβ42/Aβ40-ratio: The ratio of peripheral Aβ42 and Aβ40 levels was lower in both patients with cognitive frailty (b=-0.015 [-0.28; -0.03], *p* = 0.023) and frailty (b=-0.11 [-0.21; -0.01], *p* = 0.013), but was not significantly altered in robust patients with cognitive impairment (b = 0.00 [-0.22; 0.22], *p* = 0.986, see Fig. 3, bottom, and supplementary table S7).

Results did not change substantially when considering an inverse instead of a logarithmic link function. Likewise, analyses for Aβ42-levels, Aβ40-levels and their ratio were repeated in a subsample with Barthel score of ≥ 95 points and IADL score of 8, which also did not substantially alter the results (see supplementary tables S11-S13).

### Comparison to alternative models

#### Cortical signatures

All models including two independent variables for frailty and cognitive impairment, had slightly lower AIC (AIC_age_=-1928, AIC_AD_=-1847) and higher adjusted R² (R²_age_=0.081, R²_AD_=0.096) compared to the primary models. Although these results favor the simpler models treating frailty and cognitive impairment as independent variables and neglecting cognitive frailty as an additional exclusive group, the Davidson-MacKinnon J-test did not indicate any significant changes to the goodness of fit by using the alternative model specifications (t_age_=0.69, p_age_=0.5; t_AD_=0.43, p_AD_=0.7). In the alternative models, significant effects of cognitive impairment on the aging (b=-0.045 [-0.082; -0.090], *p* = 0.028) and AD signatures (b=-0.045 [-0.089; -0.008], *p* = 0.024) were observed, but no association with frailty (b_age_=-0.019 [-0.045; 0.006], p_age_=0.15; b_AD_=-0.017 [-0.044; 0.010], p_AD_=0.24) could be observed.

#### Plasma Aβ

The AIC slightly favored the simpler, alternative model of Aβ42/Aβ40-ratio (AIC_ratio_=-2205.5) and Aβ42 levels (AIC_Aβ42_=6994.7) over the initial models (AIC_ratio_=-2203.6, AIC_Aβ42_=6996.1). For both Aβ42/Aβ40-ratio and Aβ42 levels, D² was identical for initial and alternative models (D²_ratio_=0.215, D²_Aβ42_=0.115). Davidson-MacKinnon’s J-test did not indicate a significant difference in goodness of fit between the two models (t_ratio_=0.28, p_ratio_=0.778; t_Aβ42_=0.58, p_Aβ42_=0.56). In the reduced model, frailty (b=-0.12 [-0.21; -0.023], *p* = 0.007), but not cognitive impairment (b=-0.03 [-0.12; 0.06], *p* = 0.55) was associated with a lower Aβ42/Aβ40-ratio. For Aβ42 levels, there was an insignificant trend for lower levels in frailty (b=-0.13 [-0.27; 0.01], *p* = 0.050), but not for cognitive impairment (b = 0.01 [-0.13; 0.16], *p* = 0.91).

### Association of cortical signatures with blood-sampled biomarkers

**Fig. 4:**
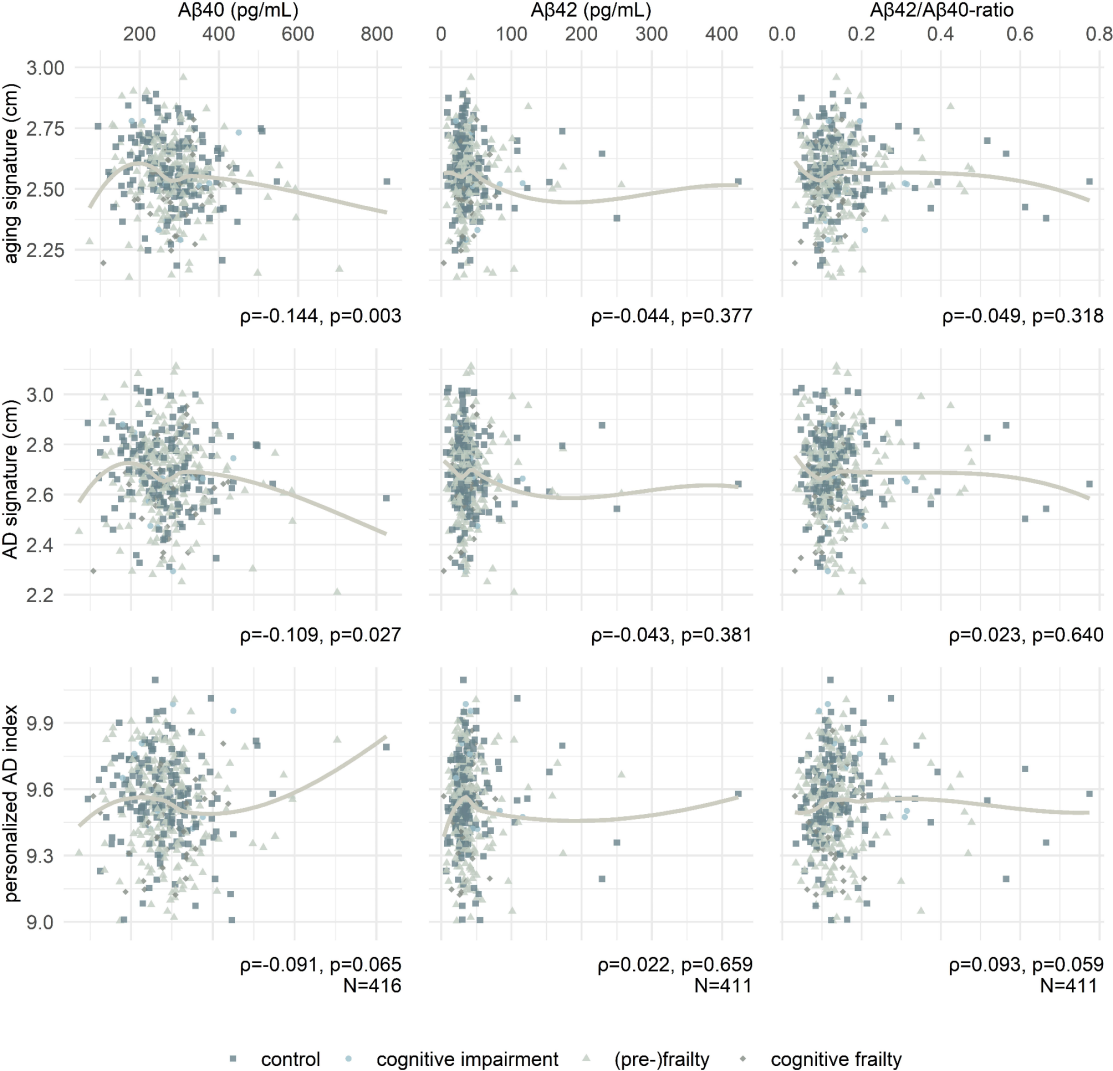
Correlations among MRI-based and blood-sampled biomarkers of aging and AD. The graphs show local regression (LOESS) lines, but effect size ρ depicted below refers to Spearman’s rank correlation coefficient

Correlations among cortical signatures and Aβ were generally weak. Higher levels of Aβ40 were associated with lower cortical thickness in aging- (ρ=-0.144, *p* = 0.003) and AD-related cortical regions (ρ=-0.109, *p* = 0.027). In addition, there was an insignificant trend for an association of a lower personalized AD index in patients with higher Aβ40 levels (ρ=-0.091, *p* = 0.065) and in patients with a lower Aβ42/Aβ40-ratio (ρ = 0.093, *p* = 0.059). A complete matrix of correlations is given in Fig. 4.

## Discussion

This work investigates the cortical atrophy patterns in relation to cognitive frailty in a clinical cohort of surgical patients. Aging- and AD-related atrophy were observed in the group of cognitively frail patients, but not in the groups with only frailty or cognitive impairment. However, this association is likely driven by a general cortical thinning associated with cognitive frailty. We further observed a lower plasma Aβ42/Aβ40-ratio in both patients with frailty and cognitive frailty, as well as lower plasma Aβ42 in frailty, suggesting a general association of amyloid β pathology and frailty rather than a specific role in cognitive frailty.

During recent years, anatomical correlates of cognitive frailty in the brain have come into the focus of neuroimaging research. The group of Zhào studied the presence of cerebral vascular damage in cognitive frailty using Fazekas scores from visual rating of MR images in 130 patients with small vessel disease [44]. Although cognitive frailty was associated with more severe stages of cerebral vascular disease, cognitive impairment was based solely on the participant’s score on a dementia screening test, which limited its validity. Wan and colleagues used T1-weighted anatomical MRI and diffusion tensor imaging to compare a sample of 26 cognitively frail, community-dwelling participants with an equal number of matched controls and reported volumetric and microstructural tissue alterations in subcortical brain regions, especially the thalamus and hippocampus, suggestive of AD-like pathology [45, 46]. Wan’s seminal study is of high methodological quality regarding the assessment of frailty and cognitive impairment, as well as the neuroimaging pipeline, but the recruited sample was small and excluded patients with isolated cognitive impairment or frailty [46]. Hence, Wan’s observations might be driven by more general associations of hippocampal volume and cognitive function in older adults and frailty [11, 15, 17, 47, 48].

Yoshiura and colleagues analyzed MRI data from 883 community-dwelling participants at risk for dementia based on a screening exam. In this sample, cognitive frailty was associated with greater vascular brain damage, but medial temporal lobe atrophy was only significant when compared to participants with neither frailty nor cognitive impairment [49]. Bearing in mind that the medial temporal lobe is a key biomarker in Alzheimer’s dementia, asymptomatic AD may contribute to the development of cognitive frailty [47]. This work provides results from the largest imaging study on patients with cognitive frailty and has a robust methodology. In contrast to Wan, this study was able to provide evidence of medial temporal lobe atrophy in cognitively frail participants, which was not observed in patients with isolated frailty or cognitive impairment. Our results are in line with Yoshiura’s findings and provide complementary evidence in a surgical cohort. Importantly, our aging signature and mean cortical thickness results indicate that various atrophy patterns may be associated with cognitive frailty, which is also supported by postmortem data published by Buchman and colleagues [9, 10]. This is further supported by the findings on Aβ40 and − 42 plasma levels and cortical atrophy: Higher levels of the former were associated with pronounced atrophy in AD- and aging-related cortical regions. A critical discussion of this method in the diagnosis of Alzheimer’s dementia is provided elsewhere [30], but in short, Aβ40 is the less pathological Aβ species and increases with age rather than being specific for AD. Therefore, our data suggests an accumulation of diverse unspecific neurodegenerative changes in cognitive frailty.

Although neither concentration of Aβ40 nor Aβ42 were altered in cognitive frailty, a lower Aβ42/Aβ40 was observed in both patients with cognitive frailty and patients with frailty, but not in patients with sole cognitive impairment. To our knowledge, no previous study has investigated peripheral plasma amyloid β species in cognitive frailty. Our results are in line with findings by Lu and colleagues, who observed an association of lower plasma Aβ42/Aβ40 ratio and incident frailty in APOE ε4 non-carriers among 477 adults ≥ 70 years participating in the Multidomain Alzheimer Preventive Trial [50]. Interestingly, we observed changes in Aβ42/Aβ40 ratios of frail and cognitively frail patients and only minor changes in absolute Aβ levels. Prominent changes in Aβ42/Aβ40 may be related to subtle, simultaneous increases of peripheral Aβ40, which may reflect an age-related increase in Aβ40 production, and slight decrease of peripheral Aβ42, possibly due to increased accumulation in brain tissue [30]. Regardless, the observed plasma amyloid trends in frail patients might be an important factor to consider when establishing an AD diagnosis.

A key point of discussion in our study is the use of ROIs derived from the previously described Desikan-Killiany atlas [26] instead of a population-derived ROI map. For most of the regions described in previous publications, corresponding regions in the DK atlas were identified. However, particularly for the aging signature, ROIs derived from DK atlas labels were considerably larger than the originally described areas [18, 20, 39], so our aging signature might be less specific for aging-related atrophy than the originally proposed biomarker. Furthermore, one region (superior frontal gyrus) included two ROIs described in both the AD and the aging signatures.

Another limitation is that our definition of cognitive frailty deviated from the I.A.N.A./I.A.G.G. consensus recommendations [3]. An alternative definition of cognitive impairment was used, as CDR assessments were not available for the BioCog cohort. Nevertheless, our definition of cognitive impairment based on both MMSE and comprehensive neuropsychological assessment facilitates comparability with other studies, especially since the MMSE has often been used to define cognitive frailty in previous studies [34, 35, 36, 44] and provides sensitivity for minor cognitive deficits in surgical patients compared to healthy controls [32, 51]. As this is a secondary analysis of a study not originally designed to evaluate cognitive frailty, there was no neurological evaluation to exclude Alzheimer’s disease, and patients with undiagnosed dementia may have also been included in the cohort. A complete neurological work-up to exclude preclinical dementia is strenuous and time-demanding and cannot be adequately evaluated in a preoperative evaluation setting, and hence this limitation is inherent to our research question.

Pre-frail patients were also included in our definition, as it has been recognized that this stage of frailty is already associated with a marked increase in vulnerability to physical stress and negative outcomes in surgical cohorts, and neuroimaging has been shown to be sensitive to changes occurring in pre-frailty [21, 29]. Nevertheless, there was a low number of patients with a fully pronounced frailty phenotype and available MRI data, which may mask an intrinsic bias: patients with advanced frailty may have been systematically excluded from MRI assessments due to chronic pain, the inability to lie quietly for the duration of the assessment or other contraindications to MRI. It is also possible that these patients might be less inclined to participate in elaborate or time-consuming clinical trials simply due to the manifestation of their frailty, i.e., exhaustion, weakness, and constraints in mobility or physical activity.

Generally, the prevalence of cognitive impairment without frailty was low, making it difficult to differentiate between alterations specific to cognitive frailty and atrophy related to an isolated cognitive impairment. Our investigation considered an alternative model neglecting cognitive frailty as an exclusive entity and treating frailty and cognitive impairment as independent variables with additive effects on cortical atrophy. These models provided a negligible improvement in goodness of fit, so that our data supports both models equally. To fill this gap, further studies with more balanced cohorts are required.

In this exploratory analysis, we do not provide data on the diagnostic performance of the chosen biomarkers, and recommendations for implementation of routine plasma Aβ measurements or preoperative MRI in surgical patients is far beyond the scope of this work. An important issue is that plasma Aβ levels have failed to reliably identify patients with Alzheimer’s disease [30, 52], and its diagnostic accuracy might also be limited in cognitive frailty. Another factor to consider is that MRI-based cortical volumetry is biased by scanner hardware and software and cannot be recommended for clinical routine. An interesting hypothesis for further studies is whether autophagy imbalance may link amyloid alterations and frailty [53, 54], as autophagy modulation may provide an efficient target to reduce postoperative complications in surgical patients [55, 56].

## Conclusion

To our knowledge, this is the first study aiming to describe specific aging- and AD-related neurodegenerative processes in cognitive frailty. In a surgical sample with low risk for dementia, atrophy in aging- and AD-related cortical regions were found to be associated with cognitive frailty. Nevertheless, these alterations are likely to reflect a more general affection of the cortex in cognitive frailty. (Pre-)frailty in general, rather than cognitive frailty, is associated with AD-like changes in plasma amyloid β concentrations. Our analysis of amyloid β species further suggests that comorbidities and unspecific accumulation of neuropathological changes drive cortical atrophy in cognitive frailty.

## Electronic supplementary material

Below is the link to the electronic supplementary material.

Supplementary Material 1

Supplementary Material 2

## Data Availability

Data from the current study is not publicly available due to constraints imposed in the consent forms. An anonymized version is available from the corresponding author on reasonable request.

## Abbreviations

AD: Alzeihmer’s Disease
AIC: Akaike’s information criterion
APOE: Apolipoprotein E
Aβ: Amyloidβ
BioCog: Biomarker Development for Postoperative Cognitive Impairment in the Elderly
CANTAB: Cambridge Neuropsychological Test Automated Battery
CDR: Clinical Dementia Rating
CF: Cognitive Frailty
CI: Cognitive Impairment
DK: Desikan-Killiany
DTI: Diffusion tensor imaging
ELISA: Enzyme-linked Immunosorbent Assay
FOV: Field-of-View
GRAPPA: GeneRalized Autocalibrating Partial Parallel Acquisition
I.A.G.G.: International Association of Gerontology and Geriatrics
I.A.N.A.: International Academy on Nutrition and Aging
MCI: Mild Cognitive Impairment
MDC: Max-Delbrück Center
MMSE: Mini-Mental State Examination
MPRAGE: Magnetization Prepared Rapid Acquisition with Gradient Echoes
MRI: Magnetic Resonance Imaging
pADi: Personalized AD index
ROI: Regions-of-Interest
TE: Time to Echo
TR: Repetition Time
