# Supplementary material for: A secondary analysis of cortical atrophy and plasma amyloid β patterns in older patients with cognitive frailty undergoing elective surgery
Source: BMC Geriatr. 2025 Jul 2;25:484. doi: 10.1186/s12877-025-05740-z (PMC12220147; doi:10.1186/s12877-025-05740-z)
Supplement: Supplementary file 1 — Supplementary Material 1 [file 12877_2025_5740_MOESM1_ESM.pdf]

SUPPLEMENT TO:

Cortical atrophy and plasma amyloid β patterns in older patients with cognitive frailty

Neuropsychological testing

Neuropsychological testing was performed by trained study assistants in accordance with a standard operating procedure, which was agreed upon by two neuropsychologists. Two independent assessors examined the data for plausibility, including the review of free-text entries of research team members. When data for a participant was incomplete, missing values were imputed for each assessment timepoint separately. If data were missing due to impaired concentration or poor understanding of test instructions, missing data were replaced with worst case imputation. When values were missing at random, e.g. due to technical difficulties or environmental disturbances, random forest imputation was applied to replace missing values for single cognitive test parameters. Missing values were imputed for participants with incomplete data sets using the missForest package for R Statistical Software was used for imputations[1]. For each cognitive variable, a random forest (growing a maximum number of 100 trees on bootstrap sampling and setting the number of randomly sampled cognitive variables at each split to the square root of assessed cognitive variables) was fit to the observed part to predict the missing part. These two steps were iterated, continuously updating the imputed matrix variable-wise, and calculating the difference between the previous imputation result and the new imputation result. The algorithm stopped once this difference increased. The out-of-bag error estimate for random forests was assessed variable-wise. Data was not imputed when neuropsychological testing was missing completely.

Simple Reaction Time (SRT): The participant is shown a square on a computer screen and asked to respond to this stimulus by selecting a button as fast as possible.

Paired Associate Learning (PAL): Boxes were displayed on the screen and opened one at a time, in a randomized order. One or more of them will contain a pattern. The patterns shown in the boxes are then displayed in the middle of the screen, once at a time, and the subject must touch the box where the pattern was originally located. Each stage had ten attempts (trials) in total (the first presentation of all the shapes, then up to nine repeat presentations). If the subject made an error, the patterns were re-presented to remind the subject of their locations. When the subject got all the locations correct, they proceeded to the next stage. If the subject could not complete a stage correctly, the test terminated.

VRM delayed recognition: The participant was shown a list of 12 words once and asked to immediately recall freely as many of the presented words as possible. Twenty minutes after the word list presentation the participant had to correctly identify the initially presented words from a 24 words list containing 12 false distractors.

GPT for the dominant hand: The participant was asked to insert 25 pegs with a key alongside into wholes in a board as quickly as possible Key slots were rotated randomly, demanding visual-motor coordination skills and manual dexterity. Test parameter of interest was the task completion time using the dominant hand. Completion times of more than 300s were removed during plausibility checks in accordance with the testing manual.

TMT: The trail making task required a subject to connect a sequence of 25 consecutive targets on a sheet of paper. There were two parts to the test: in the first, the targets were all numbers (1, 2, 3, etc.) to connect sequentially; in the second part, numbers and letters (1, A, 2, B, etc.) had to be connected in alternating order. If the subject made an error, the test administrator corrected them before the subject continued the task. The completion time taken to complete the second part of the test, in which the subject alternated between numbers and letters, was used to examine executive functions.

For the derivation of cognitive impairment, multiple cognitive test parameters were assessed and referenced to a simultaneously recruited non-surgical reference group, as previously described33. For the aggregation of the neuropsychological assessment into one dichotomous cognitive variable, we selected cognitive test parameters moderate-to-good retest-reliability in the control group[2]:

- mean correct latency from the SRT
- both number of correctly remembered items in the free recall and number of correctly recognized items after delay on the VRM
- span length in the SSP
- first trial memory score from the PAL, corresponding to the number of patterns correctly located after the first trial, summed across the completed stages
- completion time for part B of the TMT
- completion time for the GPT

Magnetic resonance imaging and determination of cortical atrophy

Imaging sequences

In Berlin, data were collected at the Berlin Center for Advanced Neuroimaging using a 3 T Magnetom Trio MR scanner (Siemens) with a 32-channel head coil. T1-weighted 3D structural brain scans were acquired using an MPRAGE sequence (magnetization prepared rapid gradient echo in 192 sagittal

slices, FOV: 256·256 mm^2, voxel size: 1·1 mm^2 at 1 mm slice thickness, TR: 2500 ms, TE: 4.77 ms, 7° flip angle). In Utrecht, data was collected with an Achieva 3 T MRI scanner (Phillips) equipped with an 8-channel head coil. For technical reasons, the scanner at this study site had to be replaced with an identical machine equipped with a 32-channel head coil during the study. A harmonized T1w GRAPPA sequence was recorded here (192 sagittal slices, FOV: 256·232 mm^2, voxel size 1·1 mm^3; at 1 mm slice thickness, TR: 7.9 ms, TE: 4.5 ms, 8° flip angle).

Determination of AD and aging signatures

The AD signature refers to the mean cortical thickness of nine cortical regions: medial temporal cortex, inferior temporal gyrus, temporal pole, angular gyrus, superior frontal gyrus, superior parietal lobule, supramarginal gyrus, precuneus, inferior frontal sulcus/middle frontal gyrus[3,4]. Here, a modified version of the AD signature was derived using the following DK atlas labels: entorhinal cortex (corresponding to the medial temporal lobe), inferior temporal gyrus, temporal pole, the inferior parietal cortex (containing the angular gyrus), the superior frontal gyrus, the superior parietal cortex, the supramarginal gyrus, precuneus and the caudal middle frontal gyrus (corresponding to the region described as inferior frontal sulcus/middle frontal gyrus).

The aging signature refers to the mean cortical thickness of eight cortical regions: calcarine cortex, caudal insula, cuneus, caudal fusiform gyrus, dorsomedial frontal cortex, lateral occipital cortex, precentral gyrus and inferior frontal gyrus[4,5]. The adapted aging signature using the DK atlas employed the following labels: pericalcarine cortex (corresponding to the calcarine cortex), insula (containing the caudal insula), the cuneus, the fusiform gyrus (including the caudal fusiform gyrus), the superior frontal gyrus (corresponding to the dorsomedial frontal cortex), the lateral occipital cortex, the precentral gyrus and partes opercularis and triangularis (in the inferior frontal gyrus).

The cortical signature was calculated as $sgn = \frac{\Sigma_{i=1}^{n} d_i \cdot a_i}{\Sigma_{i=1}^{n} a_i} = \frac{\Sigma_{i=1}^{n} v_i}{\Sigma_{i=1}^{n} a_i}$ (sgn: signature score, a= Freesurfer's Regions-of-Interest (ROI) surface area, v: Freesurfers ROI cortical volume). The pADi was defined as the ratio of the aging signature and the AD ratio scaled by a factor of 10, whereas larger values indicate stronger similarity with AD-like atrophy.

Statistical analysis: Rationale for generalized linear models

Aβ40 and Aβ42 levels, as well as the Aβ42/Aβ40-ratio were found to be heavily right-skewed. Hence, three generalized linear models were employed for Aβ40, Aβ42, and their ratio, assuming a gamma distribution with a logarithmic link function, as discussed in previous publications on analysis of right-skewed data41,42 including amyloid deposition43. In contrast to multiple linear regression, generalized linear models allow the choice of the expected distribution of the response variable, as well as a link function describing the relationship between independent and the expected values of

the dependent variable42. The gamma distribution can be used for real-valued dependent variables ranging from 0 to ∞. Whereas normal distribution assumes that variance is constant for all values of the dependent variable, gamma distribution assumes a fixed association between the expected value and variance of the dependent variable. A gamma distribution is determined by a shape parameter k and a scale parameter θ, and the association between its expected value μ and variance σ^2 is determined by scale parameter θ, since μ=kθ, and σ^2= $k\theta^2$. I.e., in our case, we expected a lower variance in Aβ levels among patients with low levels of Aβ and higher variance of Aβ levels among patients with high levels, as with increasing Aβ levels, data points became more dispersed and the data interval in the highest quartile was much wider compared to the lowest quartile. The link function is a one-to-one continuous differentiable transformation mapping the expected value μ of the response variable to the linear combination of independent variables. For the log-link, this can be written as:

$ln(\mu) = \beta_0 + \sum_{i=1}^{p} \beta_i x_i \Leftrightarrow \mu = e^{\beta_0 + \sum_{i=1}^{p} \beta_i x_i}$ [6]. Here, the logarithmic link function was chosen based on the observation that displaying the data on a logarithmic scale yielded an approximately Gaussian bell curve (see results for details on the distribution). Due to the use of a logarithm, model regression coefficients reflect ratio rather than their difference[6]. However, we repeated the analyses with the canonical inverse link function[7].

Results

SUPPLEMENTARY TABLE S1: FULL REGRESSION MODEL SPECIFICATIONS FOR THE CORTICAL AGING SIGNATURE (ADJUSTED R^2=0.080, AIC=-1928).

INDEPENDENT VARIABLE	Regression coefficient b	95% confidence interval	p-value
**COGNITIVE FRAILTY**	***-0.070***	***(-0.113; -0.028)***	***0.004****
**COGNITIVE IMPAIRMENT**	-0.024	(-0.102; 0.054)	0.527
**(PRE-)FRAILTY**	-0.017	(-0.043; 0.010)	0.238
**SEX (MALE)**	***-0.031***	***(-0.057; -0.006)***	***0.020****
**AGE (Y)**	***-0.007***	***(-0.010; -0.004)***	***<0.001****
**MRI (BETWEEN-CENTER)**	0.041	(0.001; 0.083)	0.062
**MRI (WITHIN-CENTER)**	0.019	(-0.025; 0.065)	0.423
**INTERCEPT**	3.042		

SUPPLEMENTARY TABLE S2: FULL REGRESSION MODEL SPECIFICATIONS FOR THE CORTICAL AD SIGNATURE (ADJUSTED R^2=0.095, AIC=-1847).

INDEPENDENT VARIABLE	Regression coefficient b	95% confidence interval	p-value
**COGNITIVE FRAILTY**	***-0.069***	***(-0.118; -0.020)***	***0.010****
**COGNITIVE IMPAIRMENT**	-0.033	(-0.114; 0.041)	0.412
**(PRE-)FRAILTY**	-0.015	(-0.044; 0.013)	0.318
**SEX (MALE)**	***-0.047***	***(-0.074; -0.020)***	***0.001****
**AGE (Y)**	***-0.007***	***(-0.010, -0.004)***	***<0.001***
**MRI (BETWEEN-CENTER)**	0.069	(0.027; 0.110)	0.005
**MRI (WITHIN-CENTER)**	0.014	(-0.032; 0.062)	0.589
**INTERCEPT**			

SUPPLEMENTARY TABLE S3: FULL REGRESSION MODEL SPECIFICATIONS FOR THE PERSONALIZED AD INDEX (ADJUSTED R^2=0.070, AIC=-1570).

INDEPENDENT VARIABLE	Regression coefficient b	95% confidence interval	p-value
**COGNITIVE FRAILTY**	-0.014	(-0.079; 0.050)	0.700
**COGNITIVE IMPAIRMENT**	0.030	(-0.083; 0.140)	0.572
**(PRE-)FRAILTY**	-0.006	(-0.045; 0.032)	0.749
**SEX (MALE)**	***0.050***	***(0.012; 0.088)***	***0.011****
**AGE (Y)**	-0.001	(-0.005; 0.004)	0.781
**MRI (BETWEEN-CENTER)**	***-0.086***	***(-0.148; -0.022)***	***0.006****
**MRI (WITHIN-CENTER)**	0.024	(-0.048; 0.093)	0.481
**INTERCEPT**	9.576		

SUPPLEMENTARY TABLE S4: FULL REGRESSION MODEL SPECIFICATIONS FOR MEAN CORTICAL THICKNESS (ADJUSTED R^2=0.169, AIC=-2335).

INDEPENDENT VARIABLE	Regression coefficient b	95% confidence interval	p-value
COGNITIVE FRAILTY	***-0.055***	***(-0.083; -0.027)***	***0.001****
COGNITIVE IMPAIRMENT	-0.025	(-0.075; 0.020)	0.307
(PRE-)FRAILTY	-0.013	(-0.030; 0.005)	0.172
SEX (MALE)	***-0.030***	***(-0.046; -0.013)***	***0.001****
AGE (Y)	***-0.006***	***(-0.008; -0.005)***	***<0.001****
MRI (BETWEEN-CENTER)	***0.062***	***(0.033; 0.093)***	***<0.001****
MRI (WITHIN-CENTER)	0.019	(-0.013; 0.054)	0.223
INTERCEPT	2.77		

SUPPLEMENTARY TABLE S5: FULL REGRESSION MODEL SPECIFICATIONS FOR Β-AMYLOID 40 (D^2=0.024, AIC=9105.1).

INDEPENDENT VARIABLE	Regression coefficient b	95% confidence interval	p-value
COGNITIVE FRAILTY	0.07	(-0.01; 0.15)	0.067
COGNITIVE IMPAIRMENT	-0.04	(-0.16; 0.08)	0.524
(PRE-)FRAILTY	0.01	(-0.04; 0.06)	0.650
SEX (MALE)	0.02	(-0.02; 0.07)	0.289
AGE (Y)	***0.006***	***(0.001; 0.01)***	***0.015****
BATCH	***-0.05***	***(-0.09; -0.01)***	***0.050****
INTERCEPT	-0.049		

SUPPLEMENTARY TABLE S6: FULL REGRESSION MODEL SPECIFICATIONS FOR Β-AMYLOID 42 (D^2=0.115, AIC=6996.1).

INDEPENDENT VARIABLE	Regression coefficient b	95% confidence interval	p-value
COGNITIVE FRAILTY	-0.11	(-032; 0.11)	0.294
COGNITIVE IMPAIRMENT	-0.08	(-0.37; 0.19)	0.672
(PRE-)FRAILTY	***-0.14***	***(-0.29; -0.01)***	***0.044****
SEX (MALE)	-0.03	(-0.16; 0.09)	0.597
AGE (Y)	-0.00	(-0.01; 0.01)	0.527
BATCH	0.49	(0.37; 0.60)	<0.001
INTERCEPT	3.60		

SUPPLEMENTARY TABLE S7: FULL REGRESSION MODEL SPECIFICATIONS FOR THE Β-AMYLOID 42/40-RATIO (D^2=0.215, AIC=-2203.6).

INDEPENDENT VARIABLE	Regression coefficient b	95% confidence interval	p-value
COGNITIVE FRAILTY	***-0.15***	***(-0.28; -0.03)***	***0.023****
COGNITIVE IMPAIRMENT	0.00	(-0.22; 0.22)	0.986
(PRE-)FRAILTY	***-0.11***	***(-0.21; -0.01)***	***0.013****
SEX (MALE)	-0.04	(-0.12; 0.04)	0.287
AGE (Y)	***-0.01***	***(-0.02; -0.001)***	***0.043****
BATCH	0.50	(0.46; 0.62)	<0.001
INTERCEPT	-1.80		

1. Stekhoven, D.J. & Buhlmann, P. MissForest--non-parametric missing value imputation for mixed-type data. *Bioinformatics* **28**, 112-118 (2012).
2. Feinkohl, I.*, et al.* Stability of neuropsychological test performance in older adults serving as normative controls for a study on postoperative cognitive dysfunction. *BMC Res Notes* **13**, 55 (2020).
3. Dickerson, B.C.*, et al.* The cortical signature of Alzheimer's disease: regionally specific cortical thinning relates to symptom severity in very mild to mild AD dementia and is detectable in asymptomatic amyloid-positive individuals. *Cereb Cortex* **19**, 497-510 (2009).
4. Racine, A.M., Brickhouse, M., Wolk, D.A., Dickerson, B.C. & Alzheimer's Disease Neuroimaging, I. The personalized Alzheimer's disease cortical thickness index predicts likely pathology and clinical progression in mild cognitive impairment. *Alzheimers Dement (Amst)* **10**, 301-310 (2018).
5. Bakkour, A., Morris, J.C., Wolk, D.A. & Dickerson, B.C. The effects of aging and Alzheimer's disease on cerebral cortical anatomy: specificity and differential relationships with cognition. *Neuroimage* **76**, 332-344 (2013).
6. Lindsey, J.K. & Jones, B. Choosing among generalized linear models applied to medical data. *Stat Med* **17**, 59-68 (1998).
7. Salinas Ruíz, J.M.L., Osval Antonio; Hernández Ramírez, Gabriela; Crossa Hiriart, Jose. *Generalized Linear Mixed Models with Applications in Agriculture and Biology*, (Springer International Publishing, 2023).
