# Supplementary figures and images for: A secondary analysis of cortical atrophy and plasma amyloid β patterns in older patients with cognitive frailty undergoing elective surgery
Source: BMC Geriatr. 2025 Jul 2;25:484. doi: 10.1186/s12877-025-05740-z (PMC12220147; doi:10.1186/s12877-025-05740-z)

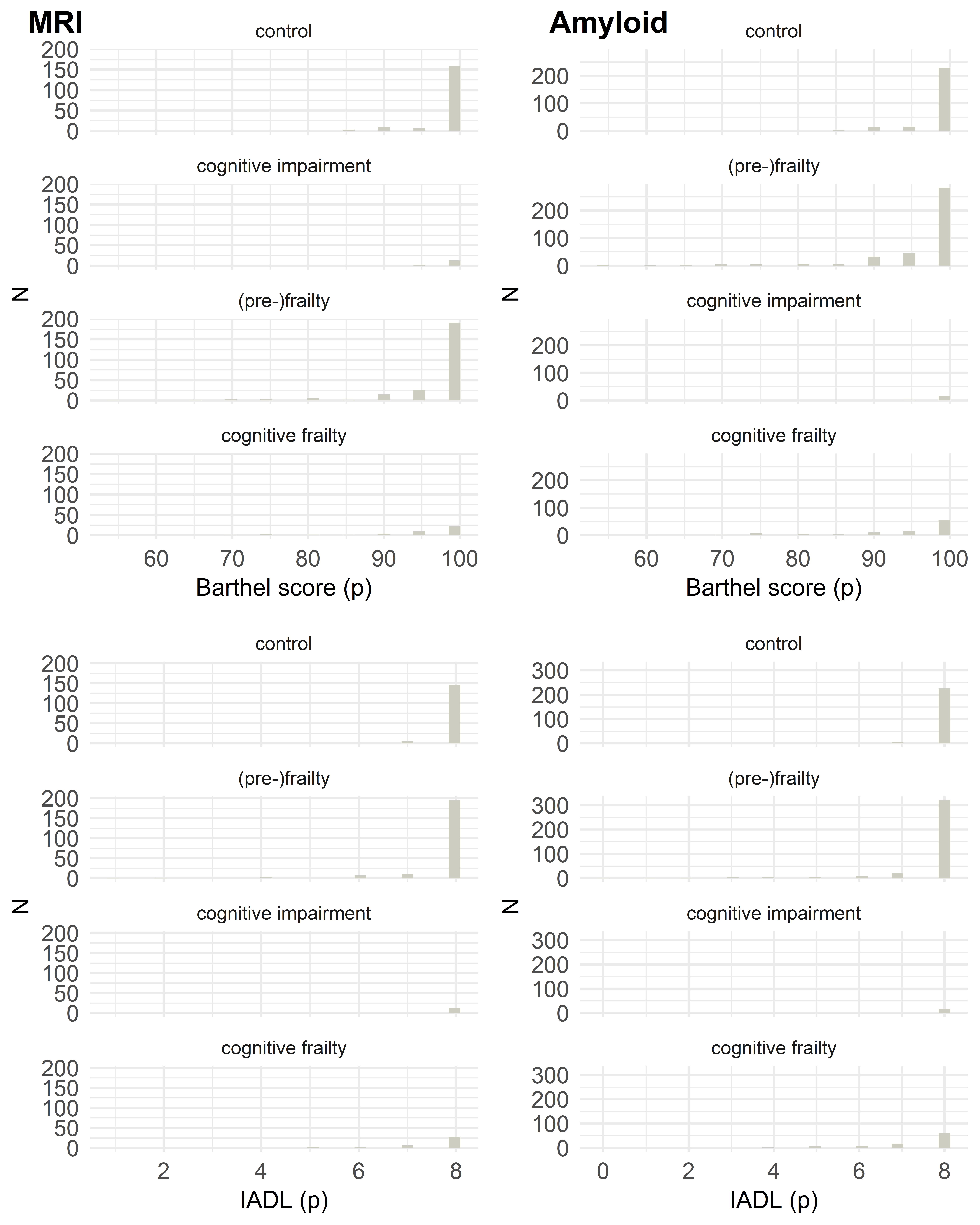

Supplement: Supplementary file 2 — Supplementary Material 2 [file 12877_2025_5740_MOESM2_ESM.jpeg]
